# Anthropometry and Body Composition Status during Ramadan among Higher Institution Learning Centre Staffs with Different Body Weight Status

**DOI:** 10.1155/2013/308041

**Published:** 2013-11-07

**Authors:** Mohd Adzim Khalili Rohin, Nurismalina Rozano, Norhayati Abd Hadi, Mohd Nasir Mat Nor, Shaharudin Abdullah, Muralidhara Dandinasivara Venkateshaiah

**Affiliations:** ^1^Faculty of Medicine and Health Sciences, Universiti Sultan Zainal Abidin (UniSZA), Kampus Kota, Jalan Sultan Mahmud, 20400 Kuala Terengganu, Terengganu, Malaysia; ^2^Kuala Terengganu Specialist Hospital, Jalan Kamaruddin, 20400 Kuala Terengganu, Terengganu, Malaysia

## Abstract

This study was done to observe the anthropometry and body composition changes before, during, and after the holy month of Ramadan. This study was carried out on 46 staff from one of the local universities, which comprised of 14 males and 32 females ranging in age from 25 to 40 years old. There were four sessions done to complete this study, namely, a week before Ramadan (T1), 1st week of Ramadan (T2), 3rd week of Ramadan (T3), and a month after Ramadan (T4). All subjects were assessed according to weight, body circumference, and body composition status. It was found that subjects with different weight status showed a significant reduction in weight (*P* < 0.01) but no significant reduction in body fat percentage (*P* < 0.05). The findings suggest that weight reduction does not promise a reduction in body fat. Changes in neck circumference were only found in normal subjects. Hence, it can be said that overweight and obese subjects showed no changes in anthropometry status during Ramadan. No changes in body composition were reported in all three weight groups except for trunk body fat. In conclusion, normal subjects showed significant changes in various anthropometry parameters, but overweight and obese subjects showed no obvious difference.

## 1. Introduction

Ramadan is the ninth month of the Islamic calendar where it is compulsory for all Muslims to abstain from eating, drinking, and smoking (just to name a few of the restrictions related to this study) from sunrise to sunset [1]. In biochemical terms it is known as abstaining from caloric intake via any route for 12–14 hours, while in Islamic terms, it stands for abstaining not only from caloric intake but also from drinking water from sunrise to sunset [2]. During the holy month of Ramadan, eligible Muslims fast for 6–10 hours a day depending on the time zone they are in and its duration varies approximately from 10 hours in winter to 18 hours in the summer [1]. 

During the nonfasting months, Muslims usually consume approximately 3-4 meals in a day. Hence, frequency and quantity of food intake may be reduced during the holy month of Ramadan. During the holy month of Ramadan, dietary intake as well as the level of physical activity is drastically reduced as most Muslims consume at least two meals per day and restrict their physical activities. Commonly, Muslims have two heavy meals during Ramadan, one just before sunrise and the other immediately after sunset or also known as the breaking of the fast [3]. Reduction in meal frequency often leads to a reduction in energy intake and a loss of body mass and fat [4–6]. El Ati et al. [7] also reported that reduction in meal intake resulted in weight loss and decrease in fat mass due to modification in calorie consumption. However, some on anthropometric status during the holy month of Ramadan showed no changes in weight. Based on a study done by Al-Hourani and Atoum [1] on 84 diabetic patients, the patients showed an increase (*P* > 0.05) in weight and BMI during the fasting period (26.86 kg/m^2^) compared to the period before the holy month of Ramadan (25.77 kg/m^2^).

Fedail et al. [8] also reported that skinfold measurements of subcutaneous body fat did not reveal any significant changes in body composition during the holy month of Ramadan. A study done by Hallak and Nomani [9] showed an average decrease in body weight during the fasting month with a total of 4.2% in boys and 3.7% in girls after 2 week of fasting. The study also revealed that about 80% of the subjects regained their lost weight within two weeks into the month of Syawal. A study done by Fedail et al. [8], Rehman and Shafiq [10], and Sayedda et al. [11] showed a reduction in weight after the holy month of Ramadan compared to before the holy month of Ramadan, while Leiper et al. [12] observed no weight reduction during the holy month of Ramadan. Lee and Nieman [13] reported that weight loss during the holy month of Ramadan is largely attributable to a reduction in fat followed by the loss of water in the body. Previous studies by Mahan and Stump [14], Mohktar and Ibrahim [15], and Poh et al. [16] also reported that waist circumference reduction was seen among subjects during the holy month of Ramadan. Consequently, this study aims to assess anthropometric and body composition status of university (UniSZA) staff during the holy month of Ramadan to ascertain if there are changes in anthropometry and body composition status as reported by previous studies or otherwise.

## 2. Materials and Methods

This study was done among 55 healthy Muslim adults aged between 25 and 40 years who were recruited for the first session of this study. However, nine subjects' dropped out from the study and only 46 subjects remained for the 2nd session until the 4th session of the study. The subjects were recruited from the UniSZA community who had responded to our invitation to take part in the study. This study was done among UniSZA staff from the Gong Badak campus between July and October 2012. A total of 46 subjects from UniSZA who had volunteered for this study had participated in this study session before, during, and after the holy month of Ramadan. The final sample size for this study was 46 subjects (14 males and 32 females) with a mean age of 33.04 ± 4.57. The nominal inclusion criteria are ages between 25 and 40 years old, apparently healthy, metabolically normal (no metabolic problems), and a Muslim who practices fasting during the holy month of Ramadan. Exclusion criteria include presence of chronic illnesses, having metabolic syndrome, on drug treatment, or being pregnant. The study protocol was approved by the Medical Research Ethics Committee, Universiti Sultan Zainal Abidin.

Anthropometry assessment for height, waist circumference (WC), neck circumference (NC), and mid-upper arm circumference (MUAC) was assessed using a portable stadiometer (SECA 217) and body circumference tape (seca203). While weight and body composition including body fat %, whole subcutaneous fat (WSF), whole skeletal muscle (WSM), trunk subcutaneous fat (TSF), trunk skeletal muscle (TSM), arm subcutaneous fat (ASF), arm skeletal muscle (ASM), leg subcutaneous fat (LSF), and leg skeletal muscle (LSM) were measured using the Karada Scan HBF-362 (OMRON). 

Subjects were advised to drink adequate water during *sahur* (the meal before sunrise) to ensure good hydration for accurate reading of body composition. All anthropometry and body composition assessments were done in four sessions, represented by T1, T2, T3, and T4. The 1st session of data collection (T1) was done a week before the holy month of Ramadan began. The second session of data collection (T2) was done a week after Ramadan (1st week of Ramadan) and the third session (T3) was done on 3rd week of Ramadan. While the last session of data collection (T4) was done a month after Ramadan (the month of Syawal). Data was collected a month after Ramadan because some subjects continued another 6 days of fasting after Ramadan had ended. Hence, to ensure that all subjects could attend the data collection sessions, the last data collection was done a month after Ramadan (end of Syawal).

## 3. Statistical Analysis

The data collected was analyzed by using Statistical Program Package for Social Sciences (SPSS) version 17.0. Descriptive statistical was performed to obtain mean and standard deviation. The level of significance was set at 0.05.

## 4. Results and Discussion 

Total of 46 subjects had participated conclusively in this study. Among the 46 subjects, 19 (41.3%) of them comprising 2 males and 17 females had a normal BMI, 18 (39.1%) of them (8 males and 10 females) were overweight and another 9 (19.6%) of them (4 males and 5 females) were obese. There were no subjects in the underweight category. All subject were Malay with a mean age of 34.3 ± 2.8 for males and 32.5 ± 5.1 for females. Mean body mass index (BMI) for males was 28.8 ± 3.4 and for females was 24.9 ± 3.5.

### 4.1. Anthropometry Changes in Different Weight Status

Anthropometry status among subjects with different weight status during the holy month of Ramadan was compared as shown in Table 1. Based on the table, all subjects showed a significant weight loss from T1 (before the holy month of Ramadan) to T4 (after the holy month of Ramadan). Therefore, compared to normal weight subjects, those who were overweight and obese showed a greater weight reduction statistically from 71.18 ± 7.13 to 70.23 ± 7.18 for overweight and from 82.56 ± 9.29 to 80.49 ± 10.84 for obese subjects. Body fat percentage of all subjects was reduced slightly, from 29.78 ± 6.13 (T1) to 28.76 ± 5.46 (T4) for normal subjects, 31.07 ± 4.49 (T1) to 30.55 ± 4.85 (T4) for overweight subjects, and 35.49 ± 4.32 (T1) to 34.77 ± 4.53 (T4) for obese subjects.

However, the slight reduction in body fat among all weight groups was not statistically significant (*P* > 0.05). Waist circumference (WC) in normal subjects showed a significant decrease before the holy month of Ramadan (72.23 ± 6.39), during the 1st week of the holy month of Ramadan (71.38 ± 6.28), and after the holy month of Ramadan (71.29 ± 6.31) with *P* < 0.001. WC on overweight and obese subjects also showed a significant difference from T1 (*P* < 0.001) with 82.13 ± 6.74 (overweight) and 91.91 ± 6.37 (obese) and on T4 (*P* < 0.05) with 83.22 ± 10.96 (overweight) and 90.62 ± 8.67 (obese). On T2 and T3, WC on overweight and obese subjects showed no significant difference statistically (*P* > 0.005). 

Mid-upper arm circumference (MUAC) status among all groups (normal, overweight, and obese) was significantly different from T1 until T4. In the normal weight group, MUAC was slightly increased from 27.15 ± 3.02 (T1) to 27.34 ± 2.86 (T2) and reduced to 26.26 ± 2.77 (T3) but increased slightly (27.02 ± 3.42) after the holy month of Ramadan (T4). In the overweight group, MUAC was reduced to 31.06 ± 1.99 (T2) compared to 31.83 ± 2.36 (T1) and increased to 31.10 ± 2.02 (T3) but reduced back to 30.99 ± 2.48 after the holy month of Ramadan (T4). MUAC in the obese weight group also increased a week into the holy month of Ramadan (T2) with 35.37 ± 2.59 compared to 34.47 ± 2.74 a week before the holy month of Ramadan (T1). However, MUAC in the obese weight group continuously reduced on T3 (34.58 ± 2.45) and T4 (34.39 ± 2.91).

Neck circumference (NC), which has recently been used as a screening tool for obesity, showed no significant difference statistically on overweight and obese weight groups. However, NC showed significant difference before, during, and after the holy month of Ramadan for normal weight group, with 33.13 ± 3.29 (T1) and then reduced to 32.55 ± 2.77 (T2). NC in the normal weight group continued to decrease on T3 (32.12 ± 2.76) but slightly increased to 32.23 ± 3.00 (T4).

Both, normal and overweight subjects showed a reduction in weight during the holy month of Ramadan and this increased slightly after the holy month of Ramadan but was still lower compared to the weight before the holy month of Ramadan. Obese subjects showed a continuous reduction of weight before the holy month of Ramadan and until a month after the holy month of Ramadan. A study done by Ramadan [17] among obese subjects and by Saada et al. [18] among overweight subjects both showed a significant weight reduction after the holy month of Ramadan, which also reflects a reduction in body composition including fat mass, muscle mass, and total water in the body. Sadiya et al. [19] had also reported that overweight subjects lose more weight during the holy month of Ramadan compared to normal and obese subjects.

There is a lot of arguments on how weight reduction is interpreted during the holy month of Ramadan. Many studies have reported that calorie intake during the holy month of Ramadan increased compared to other months. Sugary food and drinks are consumed more frequently during the holy month of Ramadan [20]. It is suggested that weight reduction during the holy month of Ramadan is due to increasing physical activity despite an increase in calorie intake during the holy month of Ramadan. Both studies by Salehi and Neghab [21] and Ramadan [17] agreed and concluded that the “Terawih” prayers that involve long periods of standing and other routines may be the reason why weight reduction is noticed during the holy month of Ramadan although the same calorie intake is present. Hence, physical activity during the holy month of Ramadan is increased due to the extended hours of praying [17]. 

There was no difference in body fat (%) before, during, and after the holy month of Ramadan among all weight groups even though some weight reduction was indicated. An increase in body fat percentage is usually coupled with an increase in weight [22]. However, reduction in weight does not indicate a reduction in body fat percentage. A study done by Leiper et al. [12] also showed no significant difference in total body fat before and after the holy month of Ramadan. Studies by Stote et al. [23] and Ramadan [17] also showed a reduction in weight but no change in body fat after the holy month of Ramadan as compared to before the holy month of Ramadan. Hence, some studies have shown a great reduction in lipid profile during the holy month of Ramadan [24, 25]. Yet, some also showed a reduction in HDL and an increase in LDL cholesterol while there is a reduction in calorie intake and no significant improvement in the lipid profile [26]. This could be the reason why body fat percentage showed no reduction with the reduction in weight.

Many studies have reported a reduction in waist circumference after the holy month of Ramadan [11, 19, 20]. In this study, WC in the normal weight group was significantly reduced after the holy month of Ramadan. However, WC in overweight and obese weight subjects showed no reduction during the holy month of Ramadan but WC in obese subjects was reduced after the holy month of Ramadan as compared to before the holy month of Ramadan. Surprisingly, WC in overweight subjects increased after the holy month of Ramadan. In overweight and obese people, waist circumference is a better indicator of distribution of adipose tissue around the abdominal region compared to waist hip ratio and BMI [5]. Chan et al. [5] also demonstrated that WC has strong correlations with subcutaneous abdominal adipose tissue mass (*r* = 0.67) compared to BMI.

### 4.2. Body Composition Status among Different Weight Groups

Body composition status is one important parameter that might change during fasting.  Table 2 shows the body composition value in subjects with different weight status. Body composition is basically muscle and body mass, which includes whole subcutaneous fat, whole skeletal muscle, trunk subcutaneous fat, trunk skeletal muscle, leg subcutaneous fat, leg skeletal muscle, arm subcutaneous fat and arm skeletal muscle. However, compared to anthropometry status, changes in body composition cannot be observed in a short period since it involves muscle and body fat. Meanwhile, anthropometry is usually associated with subcutaneous fat and muscle mass measurement, which is sometimes calculated using mathematical formulas.

Based on Table 2, mean WSF before Ramadan in the normal weight group was 24.47 ± 5.36, overweight was 25.61 ± 6.38, and obese was 30.53 ± 7.61. Both normal and obese subjects showed a slight decrease in WSF, though the reduction is not significant. Overweight subjects showed no reduction in WSF before Ramadan and until after Ramadan. In terms of WSM, normal subjects showed no difference in WSM before (25.65 ± 3.45) and during Ramadan (25.84 ± 3.14, 25.84 ± 3.16). The same goes for overweight subjects, where no significant difference was seen before Ramadan (26.89 ± 3.37), a week after Ramadan (26.87 ± 3.47), or the third week of Ramadan (26.81 ± 3.62). While, overweight subjects showed a slight increase in WSM during the third week of Ramadan (26.60 ± 4.08) compared to WSM before Ramadan (25.03 ± 3.11). However, all subjects showed an increase in WSM a month after the holy month of Ramadan but it is not statistically significant.

TSF in normal, overweight, and obese subjects showed a reduction a month after Ramadan (20.17 ± 4.63, 22.09 ± 5.13, 26.74 ± 6.05) compared to a week before Ramadan (21.21 ± 5.19, 23.06 ± 5.48, and 27.47 ± 6.16). Reduction of TSF in normal and obese subjects was significant (*P* < 0.05). TSM in obese subjects was lower compared to normal and overweight subjects. Based on the Table 2, TSM in normal and obese subjects showed a slight increase after Ramadan, with 20.65 ± 3.19 (T1) compared to a month after Ramadan (21.17 ± 3.48) in normal subjects and 17.72 ± 2.37 (T1) to 18.28 ± 2.39 (T4) in obese subjects. Overweight subjects showed no increment in TSM before, during, and after the holy month of Ramadan. Both normal and overweight subjects showed no difference in TSM during the holy month of Ramadan. Obese subjects showed an increase in TSM during the first and third week of the holy month of Ramadan.

Arm and leg regions showed higher percentage of fat compared to other body parts. Based on the results shown below (Table 2), there were no significant changes in fat found in the arms, legs, and the whole body itself. The significant changes of fat were only observed around the abdominal area among normal and obese subjects. Meanwhile, there were no significant changes seen in muscle percentage among all subjects.

Based on Table 1, all three weight groups (normal, overweight, and obese) showed no significant differences in increasing or decreasing body fat and muscle. Fat and muscle percentage was higher on the arms and legs but it was not statistically significant. Normal and obese subjects showed significant changes in TSF compared to overweight subjects. Based on the table, a change in TSF is more evident compared to changes in other body fat areas. Obese people usually have greater proportion of fat around the upper body, especially around the abdomen (trunk) compared to the thighs or hips [13]. 

Based on the study done by Al-Hourani and Atoum [1], body fat percentage and total water in the body is altered significantly during the first week of the holy month of Ramadan but muscle mass showed no significant alteration. Some studies have shown no significant changes in body fat or lean mass during or after Ramadan [7, 17]. Even using the “gold standard” technique in measuring body fat, which is computerized tomography (CT), no changes in body fat was observed during the holy month of Ramadan [25]. The study done by Yucel et al. [25] also suggests that there are no reductions in body fat during the holy month of Ramadan but there might be redistribution of fat throughout the body.

This may explain why there were significant changes in the abdominal region but no significant changes in subcutaneous fat in other parts of the body, including the arms and legs. There were no changes seen in body fat probably because once fat is gained and maintained over time, it is more difficult to lose that fat [14]. Reduction of body fat during the holy month of Ramadan, which is only a month, is not a long enough period to observe the changes in body fat in a body. 

## 5. Conclusion

Generally, there were changes in body weight, body fat percentage, and neck circumference among subjects during the holy month of Ramadan. All these three parameters showed significant reductions after Ramadan compared to a week before Ramadan. However, there were no significant reductions in waist circumference and neck circumference. Reduction in weight (*P* = 0.001) was significantly higher compared to body fat percentage (*P* = 0.03); neck circumference (NC) also showed significantly (*P* = 0.006) greater reduction after Ramadan compared to body fat. This may suggest that reduction in NC is indicative of a reduction in weight.

All subjects with different weight status showed a significant reduction in weight. Overweight subjects showed a greater reduction in body weight during the holy month of Ramadan compared to normal and underweight subjects. Normal and overweight subjects showed no difference in weight reduction. There were no significant changes in body fat observed in normal, overweight, and obese subjects during and after the holy month of Ramadan. Reduction in weight observed in subjects might be due to a reduction in total body fluids since there was no reduction in body fat. Waist circumference (WC) and mid-upper arm circumference (MUAC) showed a significant reduction after the holy month of Ramadan in all subjects in the different weight groups. However, overweight and obese subjects showed no significant changes in WC during the holy month of Ramadan; only normal subjects showed continuous changes in WC throughout Ramadan. 

Normal subjects also showed a continuous reduction in MUAC during and after the holy month of Ramadan. Overweight and obese subjects only showed a significant reduction on the first week of the holy month of Ramadan; no significant changes were seen in the third week of the holy month of Ramadan. NC in normal subjects continued to decrease throughout the holy month of Ramadan but increased slightly after Ramadan but was still less than the NC measured before the holy month of Ramadan. 

Even though there was a reduction of body fat after the holy month of Ramadan compared to before it, there was no change in body composition observed in this study. Normal and obese subjects showed a reduction in WSF, TSF, ASF, and LSF. Overweight subjects showed a reduction in TSF and ASF but no change in WSF and LSF. In terms of muscle percentage, normal subjects showed an increased in WSM, LSM, and TSM but no change in TSM. Overweight subjects only showed an increase in WSM but no changes in TSM, LSM, and ASM. Obese subjects showed no changes in ASM and LSM but showed an increase in WSM and TSM.

In conclusion, only weight reduction, WC, and MUAC were indicated after the holy month of Ramadan in all weight groups, while reduction in NC was only seen in the normal weight group. There was no improvement seen in body composition status during Ramadan in this study except TSF, even though some studies have reported otherwise. Reduction in TSF was only seen in normal and obese groups. Hence, it can be said that fasting during the holy month of Ramadan brings no significant difference in body composition. However, fasting during the holy month of Ramadan may provide improvements to general health, body structure, and spiritual enhancement if the holy month of Ramadan is celebrated modestly with moderate food intake. If one appreciates the holy month of Ramadan, the quality of life may improve to the fullest. 

## 6. Limitation

Since a high percentage of subjects that responded to our invitation were in the normal, overweight, and obese weight category, the effect on anthropometry and body composition during the holy month of Ramadan among underweight subjects could not be observed. Most of the staff of UNiSZA are already preoccupied with the dissemination of health information and are living a healthy lifestyle; hence, the probability of recruiting an underweight subject was severely reduced. More females were interested in participating in this study compared to males, which resulted in higher number of female subjects participating compared to males.

Another limitation is the period allotted to carry out this study, since the holy month of Ramadan only lasts for approximately 30 days in a year. The subjects who responded in a week after Ramadan (second session) could still be accounted for and observed but subjects who came in for the third session (third week of Ramadan) but absent during the second session and vice versa need to be excluded from study.

## Figures and Tables

**Table 1:** Various anthropometric status in subjects with different weight status.

	T1 (*n* = 46)	T2 (*n* = 46)	T3 (*n* = 46)	T4 (*n* = 46)
Weight (kg)				
Normal	53.64 ± 7.83*	53.03 ± 7.40*	52.37 ± 7.57*	52.87 ± 7.81*
Overweight	71.18 ± 7.13*	70.27 ± 7.07*	69.89 ± 7.19*	70.23 ± 7.18*
Obese	82.56 ± 9.29*	81.03 ± 10.54*	80.90 ± 9.93*	80.49 ± 10.84*
Body fat (%)				
Normal	29.78 ± 6.13**	28.94 ± 5.36	28.57 ± 5.52	28.76 ± 5.46
Overweight	31.07 ± 4.49**	30.81 ± 4.67	30.65 ± 4.63	30.55 ± 4.85
Obese	35.49 ± 4.32**	34.53 ± 4.72	34.42 ± 4.68	34.77 ± 4.53
WC (cm)				
Normal	72.23 ± 6.39*	71.38 ± 6.28*	70.05 ± 5.67*	71.29 ± 6.31*
Overweight	82.13 ± 6.74*	83.22 ± 8.60	83.44 ± 2.03	83.22 ± 10.96**
Obese	91.91 ± 6.37*	92.39 ± 8.28	90.66 ± 7.85	90.62 ± 8.67**
MUAC (cm)				
Normal	27.15 ± 3.02*	27.34 ± 2.86*	26.26 ± 2.77*	27.02 ± 3.42*
Overweight	31.83 ± 2.36	31.06 ± 1.99*	31.10 ± 2.02	30.99 ± 2.48*
Obese	34.47 ± 2.74	35.37 ± 2.59*	34.58 ± 2.45	34.39 ± 2.91*
NC (cm)				
Normal	33.13 ± 3.29*	32.55 ± 2.77*	32.12 ± 2.76*	32.23 ± 3.00*
Overweight	36.77 ± 3.40	36.53 ± 4.11	35.80 ± 3.53	36.08 ± 3.23
Obese	38.92 ± 2.78	38.50 ± 3.57	37.78 ± 2.82	37.68 ± 3.48

*Result is significant with *P* value < 0.01.

**Result is significant with *P* value < 0.05.

**Table 2:** Body composition status in subjects with different weight status.

	T1 (*n* = 46)	T2 (*n* = 46)	T3 (*n* = 46)	T4 (*n* = 46)
WSF (%)				
Normal	24.47 ± 5.36	23.89 ± 5.18	23.46 ± 5.23	23.63 ± 5.71
Overweight	25.61 ± 6.38	25.40 ± 6.31	25.23 ± 6.31	25.25 ± 6.57
Obese	30.53 ± 7.61	29.59 ± 7.76	29.46 ± 7.78	29.34 ± 7.22
WSM (%)				
Normal	25.65 ± 3.45	25.84 ± 3.14	25.84 ± 3.16	26.15 ± 3.50
Overweight	26.89 ± 3.37	26.87 ± 3.47	26.81 ± 3.62	27.55 ± 3.53
Obese	25.03 ± 3.11	25.77 ± 3.35	26.60 ± 4.08	26.42 ± 3.95
TSF (%)				
Normal	21.21 ± 5.19*	20.58 ± 4.68*	20.24 ± 4.75*	20.17 ± 4.63*
Overweight	23.06 ± 5.48	22.22 ± 5.01	22.14 ± 5.07	22.09 ± 5.13
Obese	27.47 ± 6.16*	26.86 ± 6.40*	26.72 ± 6.37*	26.74 ± 6.05*
TSM (%)				
Normal	20.65 ± 3.19	20.81 ± 2.86	20.92 ± 2.86	21.17 ± 3.48
Overweight	20.93 ± 3.88	20.38 ± 2.66	20.31 ± 3.04	20.42 ± 3.18
Obese	17.72 ± 2.37	19.32 ± 4.41	18.34 ± 2.58	18.28 ± 2.39
ASF (%)				
Normal	40.20 ± 10.01	39.72 ± 8.85	39.34 ± 8.77	38.80 ± 8.92
Overweight	38.91 ± 11.14	37.44 ± 11.32	37.50 ± 11.47	37.34 ± 11.74
Obese	42.59 ± 11.87	41.30 ± 12.35	41.57 ± 12.73	41.99 ± 12.20
ASM (%)				
Normal	29.06 ± 4.42	29.39 ± 4.06	29.47 ± 3.93	29.87 ± 4.30
Overweight	29.57 ± 6.21	29.54 ± 6.30	29.62 ± 6.20	29.16 ± 6.10
Obese	26.19 ± 5.88	26.50 ± 7.69	26.70 ± 7.76	26.81 ± 7.50
LSF (%)				
Normal	35.76 ± 7.98	35.09 ± 7.18	34.13 ± 7.16	34.36 ± 6.96
Overweight	35.74 ± 9.46	35.31 ± 9.45	35.31 ± 9.45	35.07 ± 9.75
Obese	41.79 ± 10.74	40.47 ± 11.31	40.27 ± 11.30	40.60 ± 10.42
LSM (%)				
Normal	37.98 ± 5.26	38.35 ± 4.69	38.50 ± 4.82	38.90 ± 5.04
Overweight	41.96 ± 5.50	41.78 ± 5.67	41.93 ± 5.70	41.91 ± 5.86
Obese	40.47 ± 4.93	40.93 ± 5.24	40.81 ± 5.35	40.76 ± 5.10

*Result is significant with *P* value < 0.05.
